# Epidemiology of Transfusion Transmitted Infection among Patients with *β*-Thalassaemia Major in Pakistan

**DOI:** 10.1155/2016/8135649

**Published:** 2016-07-31

**Authors:** Rizwan Ahmed Kiani, Muhammad Anwar, Usman Waheed, Muhammad Javaid Asad, Saleem Abbasi, Hasan Abbas Zaheer

**Affiliations:** ^1^Department of Biochemistry, PMAS Arid Agriculture University, Rawalpindi 46000, Pakistan; ^2^Department of Pathology and Blood Bank, Jinnah Postgraduate Medical Centre, Karachi 74200, Pakistan; ^3^Department of Pathology and Blood Bank, Shaheed Zulfiqar Ali Bhutto Medical University, PIMS, Islamabad 44000, Pakistan; ^4^ARI Research Cell, Children Hospital, Pakistan Institute of Medical Sciences, SZABMU, Islamabad 44000, Pakistan; ^5^Safe Blood Transfusion Programme, Ministry of National Health Services, Islamabad 44000, Pakistan

## Abstract

*Introduction.* Transfusion Transmitted Infections (TTIs) continue to be a major risk in transfusions in many parts of the world. The transfusion-dependent *β*-thalassaemia patients are particularly at risk of acquiring TTIs. The current study was undertaken to estimate the prevalence of TTIs in transfusion-dependent *β-*thalassaemia patients.* Material and Methods.* A cross-sectional study of 1253 multitransfused thalassaemia major patients was conducted in five different centres of Islamabad, Rawalpindi, and Karachi. The study subjects were screened for HIV, HCV, and HBV. The screening was performed at two centres: Department of Pathology, Shaheed Zulfiqar Ali Bhutto (SZAB) Medical University, and Blood Transfusion Services, Jinnah Postgraduate Medical Centre, from July to December 2015. The confirmatory screening was performed by Chemiluminescent Immunoassay (CLIA).* Results.* Out of the 1253 multiple transfused patients, 317 (25.3%) were infected with TTIs. HCV was positive in 273 cases (21.7%), HBV in 38 cases (3.0%), and HIV in 6 cases (0.5%).* Conclusion.* HCV was the leading TTI in multitransfused thalassaemia major patients in the study. Presence of HIV in thalassaemia patients is a recent disturbing development in Pakistan. Improved regulation of blood banks including use of internationally or nationally evaluated kits will bring down the incidence of TTIs in transfusion-dependent *β*-thalassaemia patients. More stringent behavioral and serological pretransfusion screening of blood for TTIs must be implemented in blood banks.

## 1. Introduction

Thalassaemias are inherited genetic disorders of haemoglobin. Different mutations in globin genes are main cause of thalassaemia worldwide [1]. Approximately 1.5% of the population is estimated to be carriers for *β*-thalassaemia with 50–60,000 new thalassaemia patients being born each year [2]. *β*-thalassaemia is mostly prevalent in population of the Mediterranean region but also found in Africa, Southeast Asia, and the Middle East [3].

The blood transfusion service in Pakistan is fragmented and largely the result of proliferation of different types of blood centres that rely on family replacement donations [4]. Reports indicate that there are over 1800 blood centres in the country with a major contribution by the private/NGOs sector. The role of the private nonprofit sector in the blood transfusion is significant, largely as a result of unmet demand of transfusion, driven mainly by the huge burden of thalassaemia disease in the country [5].

Thalassaemia is the most prevalent genetic blood disorder in Pakistan. Thalassaemia minor or carrier which is the mild form of the disorder has a prevalence of 5–7% in the country (8–10 million population). It is estimated that about 100,000 patients suffering from thalassaemia major, the severe form of the disorder, are present in Pakistan. And every year this number is increasing by 5–9,000 [6, 7]. These patients are dependent on regular blood transfusions to sustain life in addition to expensive chelation therapy and other medical managements. As a result, thalassaemia is a major healthcare challenge and places great psychological and financial stress on the affected families and is a huge burden on the national healthcare delivery system [6].

Thalassaemia gene is not randomly distributed in Pakistan and is largely restricted to the affected families. Pakistan is seeing a large increase in thalassaemic patients due to lack of any well-defined narrative or policy at the national level. According to reports, Pakistan has the highest prevalence of children with *β*-thalassaemia in the world. This is largely due to social and cultural system, the first choice is to marry within the ethnic groups, and also there is high occurrence of consanguineous marriages [8]. These factors contribute to the entrapment of the thalassaemia gene within the affected families. The provinces of KPK (Khyber Pakhtunkhwa) and Sindh have passed laws on thalassaemia prevention proposing compulsory screening for the couples intending to get married. However, the implementation of these legislations has been limited due to the cost of the test, limited ability of the laboratories to perform this expensive and complex test, and lack of implementation mechanism [9].

Transfusion Transmitted Infections (TTIs) are major risk in the developing countries. The patients of *β*-thalassaemia major are at a particularly increased risk of TTIs due to repeated transfusions. The major TT viruses of clinical importance are human immunodeficiency virus (HIV-I/II), hepatitis C virus (HCV), and hepatitis B virus (HBV) [3]. The patients depend on regular blood transfusions to sustain life. Frequent transfusions may lead to further complications such as iron overload if the patients are not properly managed medically. The transmission of HIV, HCV, and HBV is a major problem in developing countries where blood safety standards are not very high [10].

This multicentre study was conducted to shed light on the epidemiology of HIV, HCV, and HBV infection in patients with *β*-thalassaemia in Pakistan.

## 2. Material and Methods

A cross-sectional multicentre study was conducted at five different thalassaemia centres located in Rawalpindi, Islamabad, and Karachi. These centres provide screened blood and essential medical care for thalassaemia patients. The centres included Thalassaemia Centre of SZAB Medical University; Pakistan Thalassaemia Centre; Jamila Sultana Foundation Centre; Kashif Iqbal Thalassaemia Centre; and Fatimid Foundation Centre. The blood screening was performed at the Department of Blood Transfusion Services, SZAB Medical University, Islamabad, and Jinnah Postgraduate Medical Centre, Karachi, from July to December 2015. A total of 1253 blood transfusion-dependent *β*-thalassaemia patients registered at these centres were selected. The study analyzed the seroprevalence of HIV, HCV, and HBV infections in the selected population belonging to different regions of the country and ethnic groups who are residing in Rawalpindi, Islamabad, and Karachi. *β*-thalassaemia patients were receiving regular transfusions from thalassaemia centres every month to preferably maintain a haemoglobin level of 9-10 g/dL. Blood was collected from patients aseptically and serum separated in Eppendorf tubes and stored at –20°C. Screening for HIV, HBV, and HCV was performed through Chemiluminescence Immunoassay (CLIA) technique on Abbott ARCHITECT® i2000 system. CLIA is a method to determine the concentration of samples according to the intensity of the luminescence that the chemical reaction emits. The advantage of CLIA is significantly increased sensitivity and dynamic range, which allows detection of lower analyte concentrations and hence earlier diagnosis of disease.

All information and test results were kept confidential and patients were informed of a positive result through their clinician. Written consent was taken from all patients or from their relatives in case of minor for this study. The study was approved by the Institutional Review Board of the PMAS Arid Agriculture University, Rawalpindi. Inclusion criteria were known cases of *β*-thalassaemia, those who were transfused regularly, and patients of all ages and sexes. The data were entered in MS-Excel and analyzed through SPSS software. The different explanatory summaries were prepared for the important attributes of the recruited subjects. The distribution of various demographic variables was checked. Statistical significance of the random distributions was checked through Chi-square test and *t*-test at a cut-off value of *p* < 0.05.

## 3. Results

A total of 1253 subjects fulfilling the selection criteria were included in the study. The mean age of patients was 10.1 ± 6.4 years. Most of the patients 840 (67.0%) were of up to 12 years of age whereas 413 (33.0%) were between 13 and 39 years of age. In the selected population, 674 were males and 579 were females (M : F ratio = 1.2 : 1). The highest representation of subjects was from Punjab province 405 (32.3%), followed by Khyber Pakhtunkhwa 230 (18.3%). Moreover, 747 (59.6%) cases were from rural settings. A high prevalence 1020 (81.4%) of parental cousin and interfamily marriages was observed in this study.

The distribution of the TTIs was found similar according to gender. While associating age with TTIs, it was found out that older age of 13–39 years was significantly associated with TTIs when compared with younger age group up to 12 years (39.7% versus 30.7%, *p* value = 0.003) (Table 1).

Among the total 1253 study subjects, 317 (25.3%) were infected with TTIs. Of the 317 TTIs infected *β*-thalassaemia cases, HCV was found prevalent in 273 (21.7%), HBV was found in 38 (3.0%), and HIV was found prevalent in 6 (0.5%) cases. The results showed high proportion of HCV in males 159 (23.6%) compared to the female 114 (19.6%) patients; however, this difference was not statistically significant (*p* value = 0.10). Similarly, higher prevalence of HBV was found in males 21 (3.1%) than females 17 (2.9%); however, this was also statistically not significant (*p* value = 0.85). HIV was found more prevalent in females 4 (0.3%) than males 2 (0.2%); however, this difference was also not significant (*p* value = 0.42) (Table 2).

## 4. Discussion

*β*-thalassaemia is an inherited genetic disorder in which the patients need regular transfusion to sustain life [11]. Frequent blood transfusions increase the risk of transmission of infections in these patients. Transfusion Transmitted Infections have been major problem in *β*-thalassaemia patients in the developing countries due to lack of awareness and poor donor screening practices (suboptimal or deficient quality control, reliance on rapid testing in some settings, etc.) [10]. The aim of this study was to estimate the prevalence of infectious diseases like human immunodeficiency virus (HIV), hepatitis C virus (HCV), and hepatitis B virus (HBV) in transfusion-dependent *β*-thalassaemia patients.

In the current study, the average age of *β*-thalassaemia patients was 10.1 ± 6.4 years. Earlier studies show similar figures including the study by Alavi et al. which reported an average age of 11.5 ± 5.2 in *β*-thalassaemia patients [12]. Similarly, the study by Ansari et al. estimated the average age as 8.5 ± 6.42 years [13]. Sanei-Moghaddam et al. reported an average age of 9.7 ± 5.2 [14]. The ages of patients are comparable to our study findings as majority of *β*-thalassaemia is diagnosed in pediatric age.

In the present study, the results showed predominant prevalence of males (53.8%) than females (46.2%). A comparable finding (56.4%) on male dominance was reported by Mirmomen et al. [15]. Our results are also consistent with many other studies showing that the prevalence of *β*-thalassaemia disease is more common in males than females [13, 16, 17]. But there is no association reported of gender association with the genetic disease.

The current data showed dominance of consanguineous marriages (81.1%). This proved that consanguineous marriages are a major source of spread of genetic disorders including thalassaemia in our country. These results are supported by other studies as well [18–20].

The significant finding of our study was high prevalence of HCV (21.7%) cases in transfusion-dependent *β*-thalassaemia patients. The results were comparable with other studies including Jaiswal et al. who reported 21.0% cases of HCV [21]. Similarly, Jamal and colleagues reported 22.4% cases of HCV [22]. Moreover, Rahman and Lodhi reported 30% [23] HCV positive cases in beta-thalassaemia. There are studies in contrast to our study as well. Younas et al. witnessed that 42% of their *β*-thalassaemia patients had HCV [24]. Moreover Al-Sheyyab et al. reported 40.5% [25], Mansour et al. reported 40% [16], Abed found 46% [26], and Al-Hawsawi reported 40% of HCV cases [27]. These results of various studies are consistent with the present study and show that the prevalence of HCV infection is higher than other infectious diseases in *β*-thalassaemia patients.

The revealed prevalence of hepatitis B infection among *β*-thalassaemia patients in the current study was 3.0%. There are different studies which support our findings; Jamal et al. reported 2.4% cases of HBV [22], Mirmomen et al. found 1.5% cases of HBV [15], and Vidja et al. reported 2.0% cases of HBV in thalassaemia patients [28]. There is scientific evidence which is in contrast to our study results; Rahman and Lodhi reported 6.4% cases of HBV [23], Mansour et al. reported 6.5% cases of HBV [16], Kapoor et al. reported 17% cases of HBV [29], and Shah et al. found 8.4% cases of HBV in their study [30]. The studies showed high variability in results which is due to difference in the prevalence of HBV in various parts of world.

In the current study the prevalence of HIV in *β*-thalassaemia patients was estimated 0.5%. In most of the previous studies from similar sociodemographic countries, HIV has been reported negative [16, 23, 31]. However, this is one of the very few studies reporting HIV from Pakistan. Many studies from different countries have shown variable results regarding HIV prevalence in *β*-thalassaemia patients, Oza et al. reported 3.1% [32], Erich et al. reported 17% [33], Prati et al. reported 2.9% [34], and Kapoor et al. reported 0.7% [29].

In an earlier study from JPMC Blood Bank (current study centre), the seroprevalence of HCV and HBV has been reported as 7.5% and 6.2%, respectively [35]. Another study at SZAB Medical University Blood Bank (current study centre) has reported the prevalence of HCV, HBV, and HIV as 3.26%, 2.35%, and HIV 0.017% in healthy donors [36]. The prevalence of HCV and HBV in Pakistan's general population is 4.8% and 2.5%, respectively, according to a national survey by Pakistan Medical and Research Council [37].

In our study, a high prevalence of HCV (21.7%) was observed. HCV is the most prevalent infection in thalassaemia patients around the globe. Prevalence of HCV infection reported by the Pakistani authors ranges from 2.2 to 14% [38]. In the view of that, about 10 million people in Pakistan are infected with HCV. Within Pakistan, the HCV prevalence rate varies between the four provinces; prevalence rate reported in Punjab is 6.7%, in Sindh 5%, in Baluchistan 1.5%, and in Khyber Pakhtunkhwa 1.1% [39]. The patients in the study were usually transfused at the thalassaemia centres after screening of blood to detect viral, bacterial, and parasitic infections. These results indicated that the poorly screened blood transfusions are the main cause for HCV infection in thalassaemia patients. The prevalence of HBV infection is very low as compared to HCV infection. In Pakistan, there are estimated 7–9 million carriers of hepatitis B virus (HBV) with a carrier rate of 3–5% [40]. In this study we also found that two of thalassaemia patients had coinfection of HCV and HBV. HIV prevalence (0.5%) in *β*-thalassaemia patients is a sign of warning for healthcare provider as it was never considered a threat for thalassaemia patients in our country. In 2009, UNAIDS Pakistan and the National AIDS Control Programme estimated that there were around 130,000 HIV cases in Pakistan, with an overall general population prevalence of less than 0.05% [41].

The transfusion of blood remains a key intervention and has a pivotal role in patient management. The development of quality assured blood screening systems to guarantee the screening for TTIs is an essential element of blood safety. However, this has been compromised in certain instances where poor quality screening systems have resulted in transmission of TTIs to the patients, particularly multitransfused thalassaemia patients who are not admitted to the hospital and visit centre to centre for transfusion depending on availability of blood. They remain at unacceptable risk of contracting TTIs which otherwise could easily be prevented. Therefore effective screening to confirm the presence of TTIs can decrease the risk of transmission. It is pertinent to mention here that blood safety also depends on its source, and as advocated by the WHO [42], the safest source is the regular voluntary nonremunerated donors from low risk populations.

Nucleic Acid Testing is a very sensitive technique reducing the window period to minimum but is still not viable for a developing country like Pakistan due to economic costs, although a few individual centres are using NAT using pooled samples.

In our study limited variables were selected and no more detailed information is obtained from patient's clinical condition from their documents that can help the healthcare providers with a more detailed information regarding this chronic disorder.

## 5. Conclusion

According to the study findings Transfusion Transmitted Infections were alarmingly high. HCV was the leading TTI in this study and presence of HIV was also witnessed in few cases which is a recent disturbing development in the country.

The prime focus of the study is on policy makers to decide on how to change the current practices in blood banks especially those related to TTIs screening which is posing a threat to blood safety with a higher prevalence of infectious markers in transfusion-dependent thalassaemia patients.

The screening kits for TTI markers have never been evaluated and validated at a national level which has to be done. There are limitations in case of sensitivity and specificity of screening assays and therefore complete safety in terms of liberty from the infection risk might not be assured.

The need of the hour is that Pakistan should learn and benefit from the rich regional experience of eradicating thalassaemia. We need to acquire this regional experience and adopt it to our national needs and requirements and develop a National Thalassaemia Policy and Strategic Framework which should cover all aspects of thalassaemia including the availability of safe blood.

## Figures and Tables

**Table 1:** Association of patient age and gender with transfusion transmitted infections.

	TTIs (*n* = 317)	Without TTIs (*n* = 936)	*p* value
Age (years)			
Up to 12	191 (60.3%)	649 (69.3%)	0.003
13 to 39	126 (39.7%)	287 (30.7%)	
Gender			
Male	180 (56.8%)	494 (52.8%)	0.24
Female	137 (43.2%)	442 (47.2%)	

**Table 2:** Gender distribution of TTIs (HIV, HCV, and HBV) in *β*-thalassaemia patients.

	Males (*n* = 674)	Female (*n* = 579)	*p* value
HIV	2 (0.3%)	4 (0.7%)	0.42
HCV	159 (23.6%)	114 (19.6%)	0.10
HBV	21 (3.1%)	17 (2.9%)	0.85
